# Bidirectional relationship between nocturnal subjective sleep duration and cognitive performance in Chinese over 45 years old: a national representative longitudinal study

**DOI:** 10.1186/s12877-022-03468-8

**Published:** 2022-10-26

**Authors:** Chao Li, Xianying Min, Gang Cheng, Yan Yan, Zexuan Li

**Affiliations:** 1 Department of Psychiatry, National Clinical Research Center for Mental Disorders, The Second Xiangya Hospital of Central South University, 410011 Changsha, Hunan China; 2 Hunan Key Laboratory of Psychiatry and Mental Health, Mental Health Institute of Central South University, National Technology Institute on Mental Disorders, Hunan Technology Institute of Psychiatry, Hunan Medical Center for Mental Health, 410011 Changsha, Hunan China; 3 Department of Epidemiology and Medical Statistics, Xiangya School of Public Health, Central South University, 410000 Changsha, Hunan China

**Keywords:** Cognitive decline, Night sleep duration, Bidirectional effects, Random intercept cross-lagged panel models

## Abstract

**Introduction:**

Previous studies have inconsistent associations between changes in sleep duration and cognitive function and have not separated interindividual effects from intraindividual effects. This study aimed to examine the bidirectional associations between subjective night sleep duration and cognitive function while differing intraindividual and interindividual effects.

**Methods:**

A national representative sample was obtained from China Health and Retirement Longitudinal Study during 2011–2018. Night sleep duration and potential confounders were assessed and collected by questionnaires. Cognition was assessed in three categories (orientation, executive function, and memory), and z scores were used for global cognitive performance. The random intercept cross-lagged panel model was used to examine the temporal associations during 2011–2018.

**Results:**

Across 9404 participants over 45, at interindividual level, moderate (β= -0.014) and long sleepers (β= -0.06) had positive association between sleep duration and cognitive decline after adjusted covariates, but short sleepers had negative associations between sleep duration and cognitive decline (β = 0.87). At intraindividual level, prolonged sleep duration predicted better cognition for short (β= -0.021 at wave2-3; β= -0.04 at wave3-4) and moderate (β= -0.017 at wave3-4) sleepers.

**Conclusion:**

For short sleepers, longer subjective nocturnal sleep duration predicted better cognitive performance; but moderate and long sleepers showed opposite results—short and moderate sleepers with prolonged subjective nocturnal sleep duration would have later cognitive decline. Our findings tentatively suggested that an increased subjective night sleep duration and subjective long sleep duration could be regarded as useful tools for identifying middle- and old adults at higher risks of progressing to cognitive decline.

**Supplementary Information:**

The online version contains supplementary material available at 10.1186/s12877-022-03468-8.

## Introduction

Aging of population is a characteristic worldwide [1], and the number of older adults around the world is increasing dramatically [2]. China has become an aging society in the past 20 years, with 13.26% of the total population older than 60 in 2010, the number is estimated to increase to 400million by 2050 [3]. Aging is associated with adverse health outcomes, including cognitive impairment and dementia, leading to a large proportion of disability and mortality in older adults and a heavy psychosocial and economic burden for families and society [4]. Current research focuses on preventing cognitive decline through risk factor identification and modification, with growing interest in sleep duration [5].

A growing number of grounds [6–8] showed the relationship between night sleep duration and cognitive decline among adults, especially among old adults. An inverted U-shaped association between cognitive decline and sleep duration was found [7] in a review, which means that compared with moderate sleep duration, short/long sleep duration was related to a higher risk of cognitive decline. Only one study [9] reported that cognitive decline was associated with sleep disturbance in older women. Lack of study explored whether there is a bidirectional relationship between night sleep duration and cognitive decline. Previous studies presented that patients with dementia had worse sleep quality [10] or shorter night sleep duration [11], and long sleep duration is a preclinical marker associated with worse prognosis among old adults with cognitive impairment [12]. It is possible to have different bidirectional relationships between night sleep duration and cognitive decline among people with short, moderate, or long night sleep durations. Besides, dementia has a long period of preclinical stage, so it is essential not only for old adults but also for middle-aged adults to identify the population with high risk. Meanwhile, longitudinal associations between sleep duration and cognition were more robust for men than for women, according to a previous study conducted in America [13], it is vital to test the sex difference in Chinese. Previous analyses [14, 15] from China Health and Retirement Longitudinal Study (CHARLS) have explored the associations between night sleep duration and cognitive performance using data from 2011 to 2015. There is a lack of research on bidirectional associations between night sleep duration and cognition in Chinese middle- and old- adults.

Our study aimed to explore bidirectional associations between sleep duration and cognitive decline across adults over 45 years and further distinguish between interindividual and intra-individual differences because of our statistic approach. Associations are often tested at an interindividual level in practical rather than at an intraindividual level. However, intraindividual effects are important since intraindividual processes show evidence of personalized interventions, especially in psychological theories [16]. Meanwhile, conclusions at interindividual levels could not be extrapolated to intraindividual levels. For example, the fact that adults who report extreme sleep duration are also likely to report cognitive decline does not necessarily indicate that individual adults’ cognitive performance will be improved when they improve their subjective sleep duration. The random intercept cross-lagged panel model (RI-CLPM) explains time-invariant individual differences by including random intercepts and hence causes less biased estimates of the intraindividual effects than other traditional statistic approaches.

The present study hypothesized that the bidirectional relationships existed and described them as follows: (1) Increased night sleep duration would be associated with subsequent cognitive decline, (2) Cognitive decline would be related to subsequent decreasing night sleep duration. Considering the inverted U-shaped association between sleep duration and cognitive function, we assumed that the bidirectional relationships would have differences in short, moderate, and long sleepers. Potential covariates such as obesity, depressive symptoms, and physical activities [20] were included in the analysis, and we hypothesized that the bidirectional relationships would differ in these subgroups.

## Methods

This study use sample from CHARLS, which aims to represent Chinese residents aged 45 and older. Participants were randomly selected using a proportional and multistage probability sampling design [17]. Follow-up data are collected every two years from residents and their families through self-report questionnaires and interviews. The present study used data from 2011 to 2018, 4 waves in total. The study included 17,708 individuals at baseline, the response rate was 80.5%, and 13,965 participants with biomarker information in 2011. The second survey collected 15,788 individuals in 2013, the third organized 15,333 individuals in 2015, and the fourth collected 19,817 in 2018. The sample in this study was restricted to respondents aged 45 and older at baseline, who took part in the cognition test, provided self-reported sleep duration at baseline, and completed the four times follow-up (n = 9404). The details of the inclusion are presented in Supplemental Fig. 1. Each participant provided written informed consent, and the China Health and Retirement Longitudinal Study received ethical approval from the Peking University Institutional Review Board.

### Measurement

#### Sleep duration

At wave1- 4, night sleep duration was self-reported in face-to-face interviews. Participants were asked to respond to the question, “During the past month, how many hours of actual sleep did you get at night (average hours for one night)?” The duration of night sleep was divided into three groups: short (≤ 6h per day) duration, moderate (> 6 and ≤ 8 per day) duration, and long (> 8h per day) duration, according to the previous study [14]. We divided participants included into three groups: short sleepers, moderate sleepers, and long sleepers, according to the night sleep duration reported by themselves at baseline.

#### Cognition assessments

According to the previous study, the cognitive assessment was conducted in all waves and included memory, executive function, and orientation [15]. The memory assessment task comprised immediate and delayed word recall for ten unrelated words. The memory score was the sum of terms successfully recollected in the immediate and delayed word recall tasks, ranging from 0 to 20. The orientation test comprised four questions regarding the day of the week, the month, the date of the month, and the year. One point was given for each correct answer. Executive function was assessed by using the serial sevens test, in which the participant counts backward from 100 in increments of 7 (5 successive counts, with 1 point given for each correct answer), and by copying intersecting pentagons, in which the participants were asked to observe and draw a picture of 2 overlapping pentagons (3 points were given for a successful drawing and 0 points for an unsuccessful drawing). The executive score was the sum of these two tests and ranged from 0 to 8. The overall cognitive score was the sum of memory, orientation, and executive function scores. These tests’ reliability and validity have been well documented [18]. The z scores of the cognitive function test scores were generated to compare across tests based on the current cohort. This approach has been widely adopted to calculate z scores of global cognitive functions [18].

### Covariates assessments

The potential confounders included covariables that could influence individuals’ cognitive performance [19] and factors that could affect participants’ night sleep duration. According to previous studies, midlife hypertension, midlife obesity, diabetes, depression, physical inactivity, smoking, and low educational attainment were all potential risk factors for cognitive decline [20]. Based on the data collected in this cohort study, we adjusted demographic factors, lifestyle behaviors, chronic disease history, napping duration, and taking tranquilizers or sleeping pills since these covariates could influence participants’ sleep duration or cognition. The potential confounding was used for the adjusted models. At four waves, participants were asked to respond to the question “During the past month, how long did you take a nap after lunch (average minutes for one day)?” to report their afternoon napping duration. The study used Ten-item Center for Epidemiologic Studies Depression Scale short form (CES-D) for participants to evaluate their depressive symptoms [21]. With a total score of 30, each item was scored from 0 (rarely or none of the time) to 3 (most or all the time). According to prior studies, a score of 12 or higher was defined as depressive symptoms [21]. The demographic factors (age, gender, weight, and height) and the lifestyle behaviors, including smoking, physical activities (vigorous, moderate physical activities and walking), and alcohol use, were included in Supplemental Table 1. Participants reported their chronic disease history by answering the questions, “Have you been diagnosed (Hypertension/ Dyslipidemia/ Diabetes or high blood sugar/ Cancer or malignant tumor/ Chronic lung diseases/ Liver disease/ Heart attack/ Stroke/ Kidney disease/ Stomach or other digestive disease/ Emotional, nervous, or psychiatric problems/ Memory-related disease/ Arthritis or rheumatism/ Asthma) with by a doctor? -Yes/No”.

### Statistical analysis

We used SPSS 22.0 to calculate descriptive statistics. The multiple imputation is a well-established technique for analyzing datasets with missing variables when the data are missing at random [22]. We assumed that the data in this study were missing at random and used multiple imputations for missing data. Through 1000 times imputations, 5 imputed datasets were finally used for analysis. Self-reported night sleep duration and cognitive performance data from follow-ups and covariables data at baseline included physical activities, smoking, drinking, taking sleep pills, and other variables were needed to be imputed, and we created five imputed data sets in the final. Spearman rho’s coefficients were used to test the correlations between night sleep duration and cognition at four waves.

**Fig. 1:**
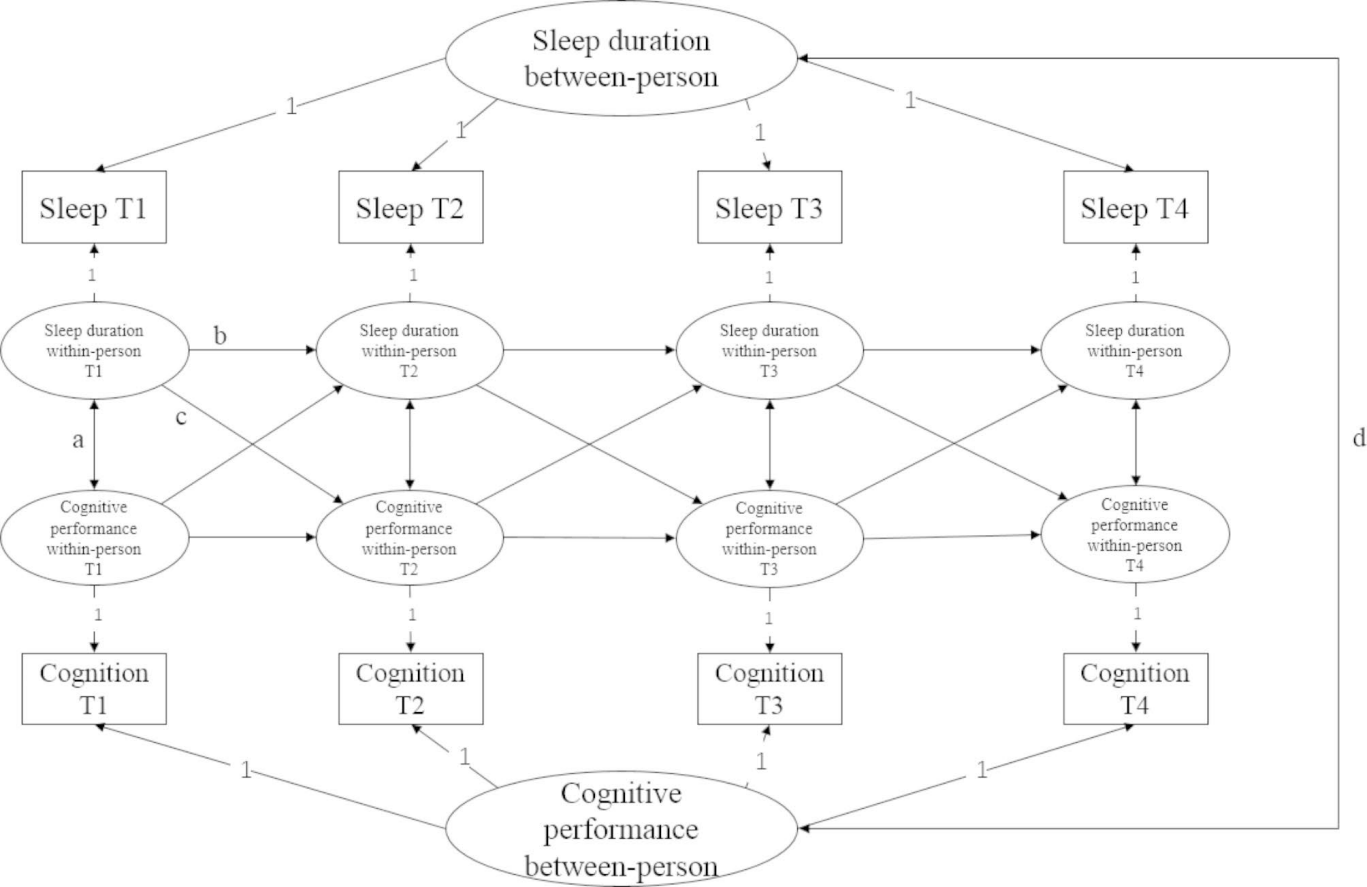
Four-waves Random Intercept Cross-Lagged Panel Model: **a**, cross-sectional paths; **b**, autoregressive paths; **c**, cross-lagged paths; **d**, correlation between stable traits of night sleep duration and cognitive performance at the interindividual level. Circles represent “latent” variables, square denote observed variables

The bidirectional relationship between nocturnal sleep duration and cognition performance was tested by using the random intercept cross-lagged panel model (RI-CLPM) (Fig. 1), since RI-CLPM overcomes the limitation of the traditional cross-lagged panel model (CLPM) and could distinguish between interindividual and intra-individual effects. We applied a RI-CLPM in which observed nocturnal sleep duration and cognitive z scores were regressed on their latent factor (constrained at one). The resulting eight latent factors were applied to identify autoregressive, cross-lagged paths, and cross-sectional associations [16]. The residual variances of the observed variables were constrained at zero. Next, we added two random intercepts (separately for nocturnal subjective sleep duration and cognition) with factor loadings denied at one into the model. The correlation between the random intercepts reflected how stable interindividual differences in sleep duration were associated with stable interindividual differences in cognition. Autoregressive paths were interpreted as to what extent prior differences from their expected values predicted intraindividual deviations in sleep duration and cognition. The cross-lagged paths showed to what extent sleep duration and cognition are linked bidirectionally. They indicated whether deviations from expected values in night sleep duration (/cognition) predicted deviations from expected values in cognition (/night sleep duration) at the next wave. Then, we applied adjusted models to adjust the possible covariates as confounding.

We also used multi-group analyses [16] to test bidirectional relationships. The factors used for multi-group studies included sleep duration, self-reported sleep quality, educational level, chronic disease history of hypertension or diabetes, smoking, depressive symptoms, overweight, and physical activity behaviors, which were regarded as risks of dementia [20] that people should pay attention. The RI-CLPM were stratified by gender to test whether there were gender differences in the bidirectional relationship between sleep duration and cognitive decline. To make the analysis more complete, we also conducted the main models using the whole data in which the participants with missing baseline variables, missing night sleep duration data, and cognitive information during Wave 2- Wave 4 were excluded. The main models also explored the associations between night sleep duration and specific cognitive domains. P false discovery rate (FDR) is used for P value adjustments of multiple comparisons.

RI-CLPM was tested with R package ‘lavaan’ [23] in R 3.5.1 using Maximum Likelihood (ML), which estimates a mean-adjusted χ^2^. Model fit indices were as follows: chi-square (χ^2^), the Comparative Fit Index (CFI), the Root Mean Squared Error of Approximation (RMSEA), Tucker-Lewis index (TLI) [24]. TLI higher than 0.95, CFI higher than 0.95, and RMSEA less than 0.08 predict a good model fit [25]. Two-tail *P* values and P FDR value less than 0.05 were considered statistically significant. Standardized coefficients were presented in this study.

## Results

### Descriptive statistics

After the multiple imputation analysis, 9404 participants were included in the study. The different characteristics of excluded and included participants are presented in Supplemental Table 2. The descriptive statistics of participants in CHARLS at baseline are shown in Supplemental Table 3 and Table 1. The sample included 9404 individuals over 45 years at baseline (46.1% male); the average night sleep duration was 6.37 (SD:1.86) hours per day at baseline. Compared with women, men were more likely to be well-educated (14.4% vs. 6.5%, P < 0.001); to smoke (70.2% vs. 44.9%, p < 0.001); to have vigorous physical activities (32.9% vs. 22.4%, P < 0.001); not to have depression symptoms (45.6% vs. 52.1%, P < 0.001); to have the chronic disease history of hypertension (20.7% vs. 24.7%, P < 0.001); to have longer night sleep durations (6.50h/d vs. 6.25h/d, P < 0.001); and to have higher cognitive performance scores (overall: 15.21 vs. 12.68, P < 0.001). Compared with participants with moderate night sleep duration, short sleepers were more likely to have shorter napping duration, while long sleepers tended to have longer napping duration (0.57h/d vs. 0.48h/d (short) vs. 0.65h/d (long), P < 0.001); short sleepers and long sleepers were more likely not to be well-educated(11.4% vs. 8.9% (short) vs. 5.5%, P < 0.001); short sleepers and long sleepers were more likely to have depressive symptoms (47.3% vs. 51.3% (short) vs. 51.6% (long), P < 0.001); short sleepers were more likely to have some chronic diseases, while long sleepers were less likely to have some chronic diseases, including chronic lung diseases, liver diseases, and heart problems.

**Table 1:** Characteristics of Participants in different night sleep duration groups in CHARLS at Baseline

Characteristic	Participants, No.(%)				P
	Total (9404)	Short^a^ (3447)	Moderate^b^ (5192)	Long^c^ (765)	
Age, mean (SD), y	58.6 (8.8)	59.57 (8.91)	57.96 (8.62)	58.69 (9.19)	< 0.001
Gender, male	4338 (46.1)	1501 (43.5)	2493 (48.0)	344 (45.0)	< 0.001
Sleep duration per night, mean (SD), h	6.37 (1.86)	4.82 (1.46)	6.91 (1.15)	9.59 (0.76)	< 0.001
Napping duration per day, mean (SD), h	0.54 (0.71)	0.48 (0.67)	0.57 (0.72)	0.65 (0.80)	< 0.001
BMI, mean (SD) ^d^	24.73 (77.47)	23.41 (4.00)	23.74 (3.94)	23.47 (4.11)	< 0.001
High level of education	951 (10.1)	308 (8.9)	591 (11.4)	52 (5.5)	< 0.001
Physical activity					
Vigorous physical activities	2560 (27.2)	945 (27.4)	1429 (27.5)	186 (24.3)	0.168
Moderate physical activities	2813 (29.9)	1022 (29.6)	1573 (30.3)	218 (28.5)	0.546
Walking	4208 (44.7)	1539 (44.6)	2340 (45.1)	329 (43.0)	0.557
Depression symptoms	4617 (49.1)	1767 (51.3)	2455 (47.3)	395 (51.6)	< 0.001
Taking sleeping pills	128 (1.4)	56 (1.6)	62 (1.2)	10 (1.3)	0.237
Current smoking	5322 (56.6)	1897 (55.0)	2978 (57.4)	445 (58.2)	0.067
Current drinking	2359 (25.1)	823 (23.9)	1350 (26.0)	184 (24.1)	0.066
Medical history					
Hypertension	2150 (22.9)	827 (24.0)	1154 (22.2)	168 (22.0)	0.133
Dyslipidemia	851 (9.0)	331 (9.6)	463 (8.9)	57 (7.5)	0.152
Diabetes	507 (5.4)	192 (5.6)	279 (5.4)	36 (4.7)	0.630
Cancer	84 (0.9)	37 (1.1)	40 (0.8)	7 (0.9)	0.341
Chronic lung diseases	911 (9.7)	401 (11.6)	458 (8.8)	51 (6.7)	< 0.001
Liver disease	372 (4.0)	175 (5.1)	179 (3.4)	18 (2.4)	< 0.001
Heart problems	1043 (11.1)	421 (12.2)	559 (10.8)	62 (8.1)	0.003
Stroke	186 (2.0)	84 (2.4)	90 (1.7)	11 (1.4)	0.038
Kidney disease	582 (6.2)	244 (7.1)	292 (5.6)	46 (6.0)	0.022
Stomach or other digestive disease	2242 (23.8)	923 (26.8)	1168 (22.5)	151 (19.7)	< 0.001
Emotional, nervous, or psychiatric problems	121 (1.3)	50 (1.5)	62 (1.2)	9 (1.2)	0.562
Memory-related disease	101 (1.1)	41 (1.2)	51 (1.0)	9 (1.2)	0.632
Arthritis or rheumatism	3332 (35.4)	1451 (42.1)	1662 (32.0)	217 (28.4)	< 0.001
Asthma	323 (3.4)	147 (4.3)	151 (2.9)	25 (3.3)	0.003
Total Score, mean (SD)	13.84 (6.31)	13.25 (6.28)	14.39 (6.25)	12.79 (6.50)	< 0.001

Note: ^a^ Short, participants with short night duration, slept < = 6h per day

^b^ Moderate, participants with moderate night duration, slept 6–8h per day

^c^ Long, participants with long night duration, slept > 8h per day

^d^ Calculated as weight in kilograms divided by height in meters squared

Supplemental Table 4 shows the correlations between night sleep durations and cognitive performance z scores in 2011–2018. Supplemental Table 5 presents the correlations between night sleep duration and each cognitive domain (orientation, executive function, and memory) performance z scores in 2011–2018. Positive relationships between cognitive performances and sleep durations were founded. Further analyses were conducted to examine these associations’ directions in a longitudinal study and explore the associations between different nocturnal sleep duration groups.

Night sleep duration and cognitive performance changes for the total sample of sex and age group at four-time points are described in Supplemental Table 6. The average cognitive raw scores were 13.84 (SD: 6.31) at wave 1, 13.66 (SD: 6.71) at wave 2, 13.07 (SD: 6.59) at wave 3, and 11.37 (SD: 7.69) at wave 4. The average cognitive z scores were 0.00007 (SD: 1.0003) at wave 1, -0.067 (SD: 1.097) at wave 2, -0.132 (SD: 1.070) at wave 3, and − 0.384 (SD: 1.182) at wave 4. Average cognitive performance scores decreased over time, but average nocturnal sleep duration did not have a clear declined trend during 2011–2018. Males had better cognitive performances and longer nocturnal sleep durations than females across time (P < 0.001).

### Random Intercept Cross-Lagged Models

#### RI-CLPM explored association between night sleep duration and cognition z score

The overall RI-CLPM model results of testing the association between night sleep duration and cognitive performance are shown in Table 2. The adjusted model’s results are shown in Supplemental Table 7.

**Table 2:** Standardized β Coefficients for the Random Intercept Cross-Lagged Panel Model Examining the Relationships Between Night Sleep duration and Cognitions^a^

	β (95% CI)			
Path	Short^c^ ( < = 6h/d)	Moderate^d^ (6-8h/d)	Long^e^ (> 8h/d)	Night sleep duration
RIns^f^~RIco^g^	**0.279 (0.257, 0.301)** ^*b^	**0.047 (0.032, 0.062)** ^*b^	**-0.078 (-0.106, -0.05)** ^*b^	**0.13 (0.115, 0.145)** ^*b^
Stability paths				
W1 sleep duration → W2 sleep duration	**-0.172 (-0.224, -0.12)** ^*b^	**-0.151 (-0.205, -0.097)** ^*b^	-0.059 (-0.275, 0.157)	-0.031 (-0.048, -0.014)
W2 sleep duration →W3 sleep duration	**0.183 (0.159, 0.207)** ^*b^	**0.268 (0.25, 0.286)** ^*b^	**0.3 (0.258, 0.342)** ^*b^	**0.087 (0.069, 0.105)** ^*b^
W3 sleep duration →W4 sleep duration	**0.208 (0.187, 0.229)** ^*b^	**0.279 (0.263, 0.295)** ^*b^	**0.316 (0.277, 0.355)** ^*b^	**0.139 (0.125, 0.153)** ^*b^
W1 cognition → W2 cognition	**-0.245 (-0.296, -0.194)** ^*b^	**-0.249 (-0.329, -0.209)** ^*b^	**-0.344 (-0.452, -0.236)** ^*b^	**-0.279 (-0.310, -0.248)** ^*b^
W2 cognition → W3 cognition	**0.115 (0.086, 0.144)** ^*b^	**0.09 (0.067, 0.113)** ^*b^	**0.124 (0.065, 0.183)** ^*b^	**0.097 (0.08, 0.116)** ^*b^
W3 cognition → W4 cognition	**0.353 (0.328, 0.378)** ^*b^	**0.358 (0.337, 0.379)** ^*b^	**0.228 (0.175, 0.281)** ^*b^	**0.344 (0.329, 0.359)** ^*b^
Cross-Lagged paths				
W1 sleep duration → W2 cognition	**0.081 (0.058, 0.104)** ^*b^	-0.019 (-0.042, 0.004)	0.064 (-0.007, 0.135)	0.016 (0.008, 0.024)
W2 sleep duration → W3 cognition	**-0.039 (-0.05, -0.028)** ^*b^	0.01 (0, 0.02)	0.021 (0.003, 0.039)	-0.002 (-0.01, 0.006)
W3 sleep duration → W4 cognition	**-0.038 (-0.047, -0.029)** ^*b^	**-0.02 (-0.027, -0.013)** ^*b^	0.013 (-0.001, 0.027)	**-0.025 (-0.031, -0.019)** ^*b^
W1 Cognition →W2 seep duration	**-0.219 (-0.412, -0.126)** ^*b^	0.077 (0.01, 0.144)	**0.523 (0.325, 0.721)** ^*b^	0.037 (-0.014, 0.088)
W2 cognition →W3 sleep duration	**-0.115 (-0.173, -0.057)** ^*b^	0.013 (-0.029, 0.055)	0.016 (-0.118, 0.15)	-0.017 (-0.051, 0.017)
W3 cognition →W4 sleep duration	**-0.285 (-0.341, -0.229)** ^*b^	-0.025 (-0.069, 0.019)	0.249 (0.116, 0.382)	**-0.103 (-0.137, -0.069)** ^*b^

Note: W1, wave 1 (CHARLS 2011 survey); W2, wave 2 (CHARLS 2013 survey); W3, wave 3 (CHARLS 2015 survey); W4, wave 4 (CHARLS 2018 survey)

^a^ Standardized coefficients are presented in these models. The subscript numbers indicate the waves of the study (wave1, wave 2, wave 3 and wave 4)

*Significant at *P* < 0.05

^b^ Significant at *P FDR* < 0.05

^c^ Short, participants with short night duration, slept < = 6h per day

^d^ Moderate, participants with moderate night duration, slept 6–8h per day

^e^ Long, participants with long night duration, slept > 8h per day

^f^ RIns, random intercept of night sleep^g^ RIco, random intercept of overall cognitive performance

^g^ RIco, random intercept of overall cognitive performance

**Fig. 2:**
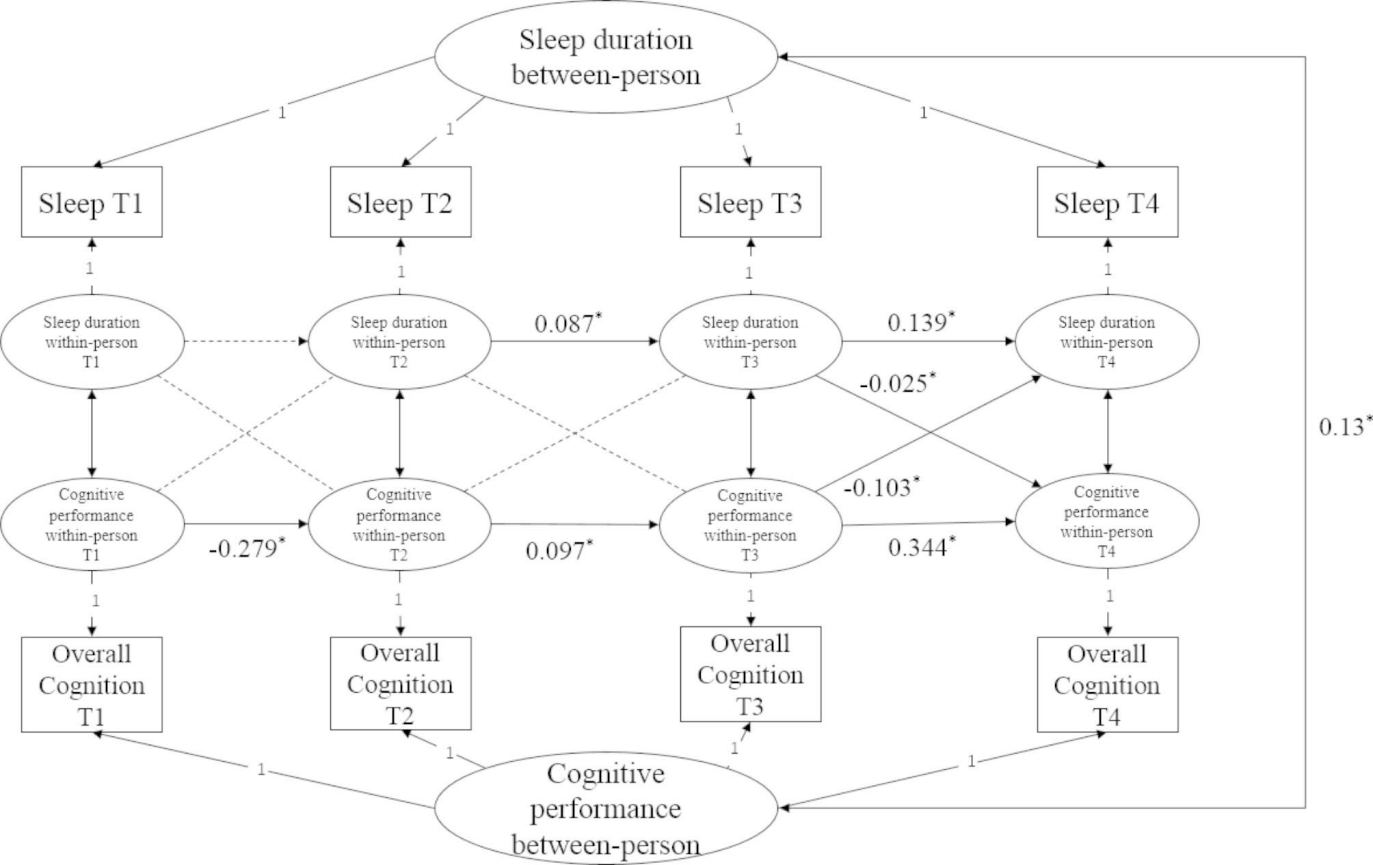
Random-intercepts cross-lagged panel model of night sleep duration and overall cognitive z score from wave 1 to wave 4 in the China Health and Retirement Longitudinal Study (n = 9404). NS = intraindividual centered night sleep duration, ns = interindividual night sleep duration, OC = intraindividual centered overall cognitive performance, oc = interindividual overall cognitive performance, RI_NS = random intercept of night sleep, RI_OC = random intercept of overall cognitive performance. Standardized estimates are presented. *P FDR < 0.05. Interindividualal association between night sleep duration and cognitive function is significant (β = 0.13). The autoregressive paths of night sleep duration and cognitive performance are significant at intraindividual levels as the solid lines in the figure, and the cross paths of night sleep duration and cognitive performance are significant at intraindividual levels as the solid lines in the figure

The associations between nocturnal sleep duration and cognitive performance were divided into interindividual and intraindividual effects, which are shown in Table 2; Fig. 2. The overall model fit of the RI-CLPM was good, χ2(1) = 130.164, P < 0.001; RMSEA = 0.038; CFI = 0.995; TLI = 0.986. At between person level, longer sleep duration is associated with better cognitive performance during the eight years (β = 0.13, P FDR < 0.001). On average, cognitive performance z scores tended to decrease at wave 4 when one slept more than their normal level of sleep duration (β= -0.025[95%CI, -0.031 to -0.019], P FDR < 0.001) at wave 3. Meanwhile, night sleep duration at wave 4 tended to decrease when participants had worse cognitive performance than their normal level of cognition z scores at wave3 (β= -0.103[95%CI, -0.137 to -0.069], P FDR < 0.001). After adjusted covariates, similar results were found.

#### Multi-group analysis of RI-CLPMs in participants with short, moderate, and long night sleep duration at baseline

The multi-group analysis of different sleep duration results showed significant differences (χ^2^ = 85.067, P FDR < 0001) in Table 2. The initial model results showed that short and moderate sleepers who reported longer sleep duration were more likely to report better cognitive function (β = 0.279; β = 0.047, P FDR < 0.005) at between person level, while long sleepers who reported longer subjective night sleep durations reported worse cognitive function as well (β= -0.078, P FDR = 0.005) at between person level. For short sleepers, results of intraindividual cross-lagged paths showed that participants with an increase in night sleep duration at wave 1 tended to have a better cognitive function at wave 2 (β = 0.081[95%CI, 0.058 to 0.104], P FDR < 0.001), however, participants with an increase in night sleep duration at wave 2/wave 3 tended to have a worse cognitive function at wave 3/wave4 (β= -0.039[95%CI, -0.05 to -0.028], P FDR < 0.001 at wave 2–3; β= -0.038[95%CI, -0.047 to -0.029], P FDR < 0.001 at wave 3–4). Moderate sleepers with an increase in subjective night sleep duration at wave 3 had a worse cognitive function at wave 4 (β= -0.02[95%CI, -0.027 to -0.013], P FDR < 0.001). Meanwhile, short sleepers with an decrease of cognitive performance at wave 1/wave 2/wave 3 had an increase of subjective night sleep duration at the next wave (β= -0.219[95%CI, -0.412 to -0.126], P FDR < 0.001; β= -0.115[95%CI, -0.173 to -0.057], P FDR = 0.005; β= -0.285[95%CI, -0.341 to -0.229], P FDR < 0.001). Long sleepers with a decrease of cognitive performance at wave 1 had an increase of subjective night sleep duration at wave 2 (β = 0.532[95%CI, 0.325 to 0.721], P FDR < 0.001). Similar results of intraindividual cross-lagged paths were found in adjusted models in Supplemental Table 6.

#### Multi-group analysis of RI-CLPMs in male and female

The results of a multi-group analysis of males and females were performed to assess the potential impact of the gender of participants on the main results in Supplemental Table 8. The overall model fit of the RI-CLPM was still good, χ^2^(1) = 158.98, P < 0.001; RMSEA = 0.041; CFI = 0.994; TLI = 0.983.The results showed similar bidirectional associations between night sleep duration and cognitive function in males. However, some associations were no longer statistically significant for females. Increased night sleep duration at wave 3 predicted worse global cognition z scores at wave 4 for females at within-person level.

#### Other multi-group analyses of RI-CLPMs

The results of separate multi-group analysis showed that the intraindividual and interindividual effects were not significantly different in various baseline factor groups such as, self-reported sleep quality levels (χ^2^ = 46.063, P = 0.121), BMI levels (χ^2^ = 42.554, P = 0.695), having chronic disease history of hypertension (χ^2^ = 20.724, P = 0.062) or diabetes (χ^2^ = 6.193, P = 0.906), having vigorous physical activities (χ^2^ = 11.469, P = 0.489), having moderate physical activities (χ^2^ = 11.106, P = 0.520), having walking (χ^2^ = 13.714, P = 0.319), smoking (χ^2^ = 6.647, P = 0.880), and having depressive symptom (χ^2^ = 17.996, P = 0.116). There were statistically significant differences in the relationships between subjective nocturnal time sleep duration and cognitive level among participants with different levels of education (χ^2^ = 25.113, P = 0.014) according to the results of the multi-group analysis Supplemental Table 9. Supplemental Table 10 presented the associations between night sleep duration and specific cognitive domains. The results showed similar bidirectional associations between night sleep duration and specific cognitive domains, including memory and executive function. But night sleep duration at Wave 3 negatively associated with orientation performances at Wave 4. The main models’ results tested using data from 3107 participants without missing variables were described in Supplemental Table 11.

## Discussion

The results showed bidirectional associations between subjective sleep duration and cognitive decline and distinguished intraindividual and interindividual differences. At interindividual levels, we found participants who reported longer sleep duration was likely to report better cognitive performance than people with shorter night sleep duration. At intraindividual levels, cognition z scores tended to decrease when one’s sleet duration increased at night than their usual level of sleep duration, and vice versa. The relationships between night sleep duration and cognitive decline were not the same at interindividual levels and intaindividual levels, since the participants in different sleep duration groups at baseline could have different associations and changes in associations. Multi-group analysis results of gender and sleep duration categories showed various associations between changes in night sleep duration and cognitive decline among different groups.

Since a previous study [7] has indicated an inverted U-shaped association between sleep duration and cognitive decline, the results of multi-group analysis on short, moderate, and long night sleep duration are necessary for further descriptions of the association. On average, short sleepers at baseline who reported longer sleep duration were more likely to report better cognitive performance than people with shorter night sleep duration. However, moderate and long sleepers at baseline who reported longer sleep duration tended to report worse cognitive performance than people with shorter night sleep duration. This is along with the results from other studies [15, 26] and meta-analyses [7], which suggested that there have been lower risks among people sleeping seven hours per day and more significant risk in people having shorter/longer sleep durations. Even the underlying mechanisms remain unclear, while cortical thinning [27], inflammations [28], and Tau proteins [29] are plausible biological pathways for explaining the association between extreme night sleep duration and cognitive decline.

The relationships between night sleep duration and cognitive decline were inconsistent in short, moderate, and long sleepers at intraindividual levels. The result of waves 1–2 presented that cognition z scores tended to increase when a shorter sleeper slept longer than their usual level of night sleep duration, which was the same as Hua et al. 2020 [14]. However, the results of waves 2–4 showed that cognition z scores tended to be lower when a shorter sleeper slept longer than their usual level of night sleep duration. Similar results were found at waves 3–4 among moderate sleepers. This is probably because that change in sleep duration at within person level could disrupt the circadian rhythms for themselves, contributing to cognitive decline since it could cause circadian dysfunction [30, 31]. There is a bidirectional relationship between homeostatic regulation of sleep duration and circadian rhythm[32]. An increase in sleep duration can disrupt the circadian rhythm by increasing daytime sleepiness, which could be at risk of neurodegenerative diseases, including dementia [29]. Moreover, clinical studies have shown more evidence that improving circadian rhythm might reduce human cognitive decline [33]. People tended to have shorter night sleep duration when a short sleeper had a lower cognition z score than their normal level of cognition. Our finding suggested that the association between cognitive decline and prolonged nocturnal sleep duration may be bidirectional for short sleepers. Possible mechanisms are that they share a common underlying etiology like neurodegeneration associated with AD or other dementias. People tended to have shorter night sleep duration when a long sleeper had lower cognition z score than his or her normal level of cognition at wave 1–2. Based on the baseline data in our analysis, long sleepers could be representative of a special group with a high risk of cognitive decline [34], since long sleepers tended to have low levels of education and high rates of depression. Mechanisms underlying long sleepers in early dementia might be related to neurodegenerative changes in brain regions involved in sleep regulation [35], which leads to circadian dysfunction and misalignment, and may result in a decrease in night sleep [36]. In this case, night sleep duration could be used as a risk marker when identifying adults who tended to have a cognitive decline in middle-aged and old- adults. People with long sleep duration are more likely to experience cognitive decline with aging. For moderate sleepers, an increase in their night sleep duration with age may lead to cognitive decline two years later.

Males and females had different night sleep duration and cognitive performance at baseline and over time. Males showed longer average night sleep durations and better cognitive performances both at the baseline and over time. According to the baseline data, males and females showed different characteristics. Males were more likely to have chronic disease history of memory-related diseases, to smoke, drink or have vigorous physical activities. However, females were more likely to have a disease history of Emotional, nervous, or psychiatric problems, to have higher rates of depressive symptoms, or to have moderate physical activities. Previous studies found that emotional symptoms related to poorer cognitive performance, and middle-aged women reported an increased risk of anxiety and depression after menopause onset [37]. The impacts of hormonal transitions on women’s sleep during their menopause and post menopause could be a possible cause of women’s shorter sleep duration and worse sleep quality than males [13]. A previous study found a stronger association between sleep deficiency and cognitive decline in males [38], which is consistent with the results of cross-lagged paths among males and females at waves 3–4 in our analysis.

A differential association between night sleep duration and specific cognitive domains, and memory impairment is the core symptom of dementia [39]. Among individuals living in the community, the decline of logical memory strongly predicts Alzheimer’s disease [40, 41]. Our results showed similar bidirectional associations between night sleep duration and cognitive performance among overall cognitive performance and memory. Systematic inflammation was a possible mechanism underlying the relation between longer sleep duration and cognitive decline [42].

Along with previous evidence [19], multi-group analysis proved that educational levels were a possible factor that could influence night sleep duration and cognitive decline. Based on that results, educational levels might be related factors that could be used for multidomain interventions for dementia prevention and an element used for finding high-risk groups of dementia.

Our study has several strengths, including the analytic approach and the large, longitudinal sample. Neurocognitive tasks used to test participants’ cognitive performance were the same for every wave, which facilitated the comparing analysis across time. The cohort with enough follow-up years and lag allow us to observe how the reciprocal relationships unfolded gradually over time under free-living conditions. Another strength was the statistic approach called RI-CLPM, which allowed us to examine changes over time on an intraindividual level since it could split variance between- and intraindividual levels.

The present results could have selective bias by excluding participants without finishing all the follow-ups, limiting the current results’ representativeness. Night sleep duration was assessed by self-reported questionnaires in this study, which could cause inaccuracies and biases in data compared to sleep data from objective measurements. Meanwhile, the cognitive development information was tested by idiosyncratic combinations of validated cognitive tasks instead of a standardized cognitive battery, which could also result in biases. The characteristics of short/moderate/long sleepers differed at baseline, even though we conducted on adjusted models to reduce the biases. However, this could suggest a possibility of bias. Although we adjusted for numerous confounding factors in our analyses, unmeasured covariates might still lead to confounding bias, including sleep disorders and APOE status. Besides, it is hard to avoid the reverse causality since they were based on a follow-up of 8 years, and further studies with a follow-up of > 10 years are necessary for the future.

## Conclusion

In conclusion, our research proves that night sleep duration and cognitive performance have an inverted U-shaped association at an interindividual level. Improvement of night sleep duration was related to cognitive decline among short and moderate sleepers, and cognitive decline indicated the later increased night sleep duration in short sleepers. However, for long sleepers, cognitive decline predicted later decreased night sleep duration. Increased night sleep duration and long sleep duration could be regarded as valuable tools for identifying middle- and old adults at higher risks of progressing to cognitive decline.

## Electronic supplementary material

Supplemental Table 1 Definitions of variables used
Supplemental Table 2 Comparison of Baseline Demographics of Populations Included and Not Included in the Study
Supplemental Table 3 Characteristics of Participants in CHARLS at Baseline
Supplemental Table 4 Correlations of night sleep duration with cognitive performance in Chinese at each time point from 2011 to 2018
Supplemental Table 5 Correlations of night sleep duration with each cognitive domain performance in Chinese at each time point during 2011-2018
Supplemental Table 6 Levels of cognitive performance and sleep duration in Chinese participants at each time point
Supplemental Table 7 Adjust β Coefficients for the Random Intercept Cross-Lagged Panel Model Examining the Relationships Between Night Sleep duration and Cognitionsa
Supplemental Table 8. Standardized β Coefficients for the Random Intercept Cross-Lagged Panel Model Examining the Relationships Between Night Sleep Duration and Cognitions in male and femalea
Supplemental Table 9. Standardized β Coefficients for the Random Intercept Cross-Lagged Panel Model Examining the Relationships Between Night Sleep Duration and Cognitions in different educational levelsa
Supplemental Table 10. Standardized β Coefficients for the Random Intercept Cross-Lagged Panel Model Examining the Relationships Between Night Sleep Duration and Cognitions in specific cognitive domainsa
Supplemental Table 11 Standardized β Coefficients for the Random Intercept Cross-Lagged Panel Model Examining the Relationships Between Night Sleep duration and Cognitions by using complete data from participants without missing variablesa
Supplemental Figure 1. Flow chart of participant selection for this study

## Data Availability

All data generated or analyzed during this study are included in this article.
