# Supplementary material for: Bidirectional relationship between nocturnal subjective sleep duration and cognitive performance in Chinese over 45 years old: a national representative longitudinal study
Source: BMC Geriatr. 2022 Oct 26;22:823. doi: 10.1186/s12877-022-03468-8 (PMC9608904; doi:10.1186/s12877-022-03468-8)
Supplement: Supplementary file 1 — Supplemental Table 1 Definitions of variables used Supplemental Table 2 Comparison of Baseline Demographics of Populations Included and Not Included in the Study Supplemental Table 3 Characteristics of Participants in CHARLS at Baseline Supplemental Table 4 Correlations of night sleep duration with cognitive performance in Chinese at each time point from 2011 to 2018 Supplemental Table 5 Correlations of night sleep duration with each cognitive domain performance in Chinese at each time point during 2011-2018 Supplemental Table 6 Levels of cognitive performance and sleep duration in Chinese participants at each time point Supplemental Table 7 Adjust β Coefficients for the Random Intercept Cross-Lagged Panel Model Examining the Relationships Between Night Sleep duration and Cognitionsa Supplemental Table 8. Standardized β Coefficients for the Random Intercept Cross-Lagged Panel Model Examining the Relationships Between Night Sleep Duration and Cognitions in male and femalea Supplemental Table 9. Standardized β Coefficients for the Random Intercept Cross-Lagged Panel Model Examining the Relationships Between Night Sleep Duration and Cognitions in different educational levelsa Supplemental Table 10. Standardized β Coefficients for the Random Intercept Cross-Lagged Panel Model Examining the Relationships Between Night Sleep Duration and Cognitions in specific cognitive domainsa Supplemental Table 11 Standardized β Coefficients for the Random Intercept Cross-Lagged Panel Model Examining the Relationships Between Night Sleep duration and Cognitions by using complete data from participants without missing variablesa Supplemental Figure 1. Flow chart of participant selection for this study [file 12877_2022_3468_MOESM1_ESM.docx]

Supplemental files1

Supplemental Table 1 Definitions of variables used.

Supplemental Table 2 Comparison of Baseline Demographics of Populations Included and Not Included in the Study

Supplemental Table 3 Characteristics of Participants in CHARLS at Baseline

Supplemental Table 4 Correlations of night sleep duration with cognitive performance in Chinese elderly persons at each time point from 2011 to 2018.

Supplemental Table 5 Correlations of night sleep duration with each cognitive domain performance in Chinese elderly persons at each time point during 2011-2018.

Supplemental Table 6 Levels of cognitive performance and sleep duration in elderly Chinese participants at each time point.

Supplemental Table 7 Adjust β Coefficients for the Random Intercept Cross-Lagged Panel Model Examining the Relationships Between Night Sleep duration and Cognitions^a^.

Supplemental Table 8. Standardized β Coefficients for the Random Intercept Cross-Lagged Panel Model Examining the Relationships Between Night Sleep Duration and Cognitions in male and female^a^.

Supplemental Table 9. Standardized β Coefficients for the Random Intercept Cross-Lagged Panel Model Examining the Relationships Between Night Sleep Duration and Cognitions in different educational levels^a^.

Supplemental Table 10. Standardized β Coefficients for the Random Intercept Cross-Lagged Panel Model Examining the Relationships Between Night Sleep Duration and Cognitions in specific cognitive domains^a^.

Supplemental Table 11 Standardized β Coefficients for the Random Intercept Cross-Lagged Panel Model Examining the Relationships Between Night Sleep duration and Cognitions by using complete data from participants without missing variables^a^

Supplemental Figure 1. Flow chart of participant selection for this study.

Table 1 Definitions of variables used

| Baseline characteristics | Wave | Definition |
| --- | --- | --- |
| Time-invariant | Wave 1 (2011-2012) |  |
| Age |  | Continuous |
| Gender |  | Male=1, Female=2 |
| BMI |  | Continuous |
| BMI groups |  | BMI<18.5 (small BMI group) = 1; 18.5≤BMI<24 (normal BMI group) = 2; 24≤BMI<28 (overweight BMI group) = 3; 28≤BMI<32 (obesity BMI group) = 4; BMI≥32 (high BMI group) = 5 |
| High level of education |  | Low levels of education (without high school education or further study) =0, high levels of education (high school or more) =1 |
| Smoking |  | Yes = 1, no = 2 |
| Current alcohol use |  | Drinking at least once per week over the previous year = 1, no = 2 |
| Hypertension |  | Yes = 1, no = 2 |
| Dyslipidemia |  | Yes = 1, no = 2 |
| Diabetes or high blood sugar |  | Yes = 1, no = 2 |
| Cancer |  | Yes = 1, no = 2 |
| Chronic lung diseases |  | Yes = 1, no = 2 |
| Liver disease |  | Yes = 1, no = 2 |
| Heart problems |  | Yes = 1, no = 2 |
| Stroke |  | Yes = 1, no = 2 |
| Kidney disease |  | Yes = 1, no = 2 |
| Stomach or other digestive disease |  | Yes = 1, no = 2 |
| Emotional, nervous, or psychiatric problems |  | Yes = 1, no = 2 |
| Memory-related disease |  | Yes = 1, no = 2 |
| Arthritis or rheumatism |  | Yes = 1, no = 2 |
| Asthma |  | Yes = 1, no = 2 |
| Blood pressure |  | Continuous |
| Vigorous physical activities |  | More than 10 minutes per week = 1, less than 10 minutes per week = 0 |
| Moderate physical activities |  | More than 10 minutes per week = 1, less than 10 minutes per week = 1 |
| Walking |  | More than 10 minutes per week = 1, less than 10 minutes per week = 2 |
| Depression |  | without depression symptoms =0, defined as depression symptoms =1 |
| Self-reported sleep quality |  | "My sleep was restless" happened: rarely or none of the time (<1d day) =1,  some or little of the time (1-2 days) =2, occasionally or a moderate amount of the time (3-4 days) =3, most or all of the time (5-7 days) =4 |
| Napping |  | Continuous |
| Time-variant | Wave 1-4 (2011-2019) |  |
| Cognitive performance score |  | Continuous variable ranging from 0 to 31 |
| Night sleep duration |  | Continuous |

Abbreviation: BMI, Body mass index

Supplemental Table 2 Comparison of Baseline Demographics of Populations Included and Not Included in the Study^a^

| Characteristic | Excluded participants | Included participants | P |
| --- | --- | --- | --- |
| Age, mean (SD), y | 61 (12) | 59 (9) | <0.001 |
| High level of education | 538 (11.5%) | 954 (10.3%) | <0.001 |
| Physical activity |  |  |  |
| Vigorous physical activities | 559 (29.4%) | 1537 (38.8%) | <0.001 |
| Moderate physical activities | 957 (50.3%) | 2418 (61.1%) | <0.001 |
| Walking | 1455 (76.7%) | 3227 (81.7%) | <0.001 |
| Depression symptoms | 2168 (46.2%) | 3649 (39.3%) | <0.001 |
| Taking sleeping pills | 28 (0.6%) | 39 (0.4%) | 0.011 |
| Current smoking | 1351 (74.1%) | 2838 (78.7%) | <0.001 |
| Current drinking | 1433 (30.6%) | 3060 (33.0%) | <0.001 |
| Medical history |  |  |  |
| Hypertension | 1267 (27.2%) | 2103 (22.8%) | <0.001 |
| Dyslipidemia | 414 (9.0%) | 827 (9.1%) | 0.64 |
| Diabetes | 296 (6.4%) | 500 (5.4%) | 0.131 |
| Cancer | 53 (1.1%) | 88 (1.0%) | 0.524 |
| Chronic lung diseases | 582 (12.5%) | 892 (9.7%) | <0.001 |
| Liver disease | 186 (4.0%) | 362 (3.9%) | 0.09 |
| Heart problems | 607 (13.0%) | 1022 (11.1%) | 0.007 |
| Stroke | 159 (3.4%) | 162 (1.8%) | <0.001 |
| Kidney disease | 319 (6.9%) | 572 (6.2%) | 0.099 |
| Stomach or other digestive diseases | 1022 (21.9%) | 2204 (23.8%) | 0.053 |
| Emotional, nervous, or psychiatric problems | 75 (1.6%) | 106 (1.1%) | 0.174 |
| Memory-related disease | 114 (2.4%) | 90 (1.0%) | <0.001 |
| Arthritis or rheumatism | 1528 (32.7%) | 3277 (35.4%) | 0.002 |
| Asthma | 220 (4.7%) | 319 (3.5%) | 0.004 |

^a^ The sums of the participants were not the same since the data included in this table with missing data

Supplemental Table 3 Characteristics of Participants in CHARLS at Baseline

| Characteristic | Participants, No. (%) | | | P |
| --- | --- | --- | --- | --- |
|  | Total (9404) | Men (4338) | Women (5066) |  |
| Age, mean (SD), y | 58.6 (8.8) | 59.1 (8.7) | 58.2 (8.9) | <0.001 |
| Sleep duration per night, mean (SD), h | 6.37 (1.86) | 6.50 (1.76) | 6.25 (1.93) | <0.001 |
| Napping duration per day, mean (SD), h | 0.54 (0.71) | 0.64 (0.74) | 0.46 (0.68) | <0.001 |
| BMI^a^ | 24.73 (77.47) | 25.49 (113.97) | 24.07 (4.19) | <0.001 |
| High level of education | 951 (10.1) | 624 (14.4) | 327 (6.5) | <0.001 |
| Physical activity |  |  |  |  |
| Vigorous physical activities | 2560 (27.2) | 1426 (32.9) | 1134 (22.4) | <0.001 |
| Moderate physical activities | 2813 (29.9) | 1229 (28.3) | 1584 (31.3) | 0.002 |
| Walking | 4208 (44.7) | 1943 (44.8) | 2265 (44.7) | 0.954 |
| Depression symptoms | 4617 (49.1) | 1980 (45.6) | 2637 (52.1) | <0.001 |
| Taking sleeping pills | 128 (1.4) | 42 (1.0) | 86 (1.7) | 0.003 |
| Current smoking | 5322 (56.6) | 3047 (70.2) | 2275 (44.9) | <0.001 |
| Current drinking | 2359 (25.1) | 1995 (46.0) | 364 (15.4) | <0.001 |
| Medical history |  |  |  |  |
| Hypertension | 2150 (22.9) | 897 (20.7) | 1253 (24.7) | <0.001 |
| Dyslipidemia | 851 (9.0) | 366 (8.4) | 485 (9.6) | 0.6 |
| Diabetes or high blood sugar | 507 (5.4) | 203 (4.7) | 304 (6.0) | 0.005 |
| Cancer | 84 (0.9) | 23 (0.5) | 61 (1.2) | 0.001 |
| Chronic lung diseases | 911 (9.7) | 481 (11.1) | 430 (8.5) | <0.001 |
| Liver disease | 372 (4.0) | 187 (4.3) | 185 (3.7) | 0.114 |
| Heart problems | 1043 (11.1) | 390 (9.0) | 653 (12.9) | <0.001 |
| Stroke | 186 (2.0) | 92 (2.1) | 94 (1.9) | 0.442 |
| Kidney disease | 582 (6.2) | 305 (7.0) | 277 (5.5) | 0.002 |
| Stomach or other digestive disease | 2242 (23.8) | 947 (21.8) | 1295 (25.6) | <0.001 |
| Emotional, nervous, or psychiatric problems | 121 (1.3) | 41 (0.9) | 80 (1.6) | 0.009 |
| Memory-related disease | 101 (1.1) | 60 (1.4) | 41 (0.8) | 0.01 |
| Arthritis or rheumatism | 3332 (35.4) | 1336 (30.8) | 1996 (39.4) | <0.001 |
| Asthma | 323 (3.4) | 175 (4.0) | 148 (2.9) | 0.004 |
| Overall cognitive score, mean (SD) | 13.84 (6.31) | 15.21 (5.83) | 12.68 (6.47) | <0.001 |

Note: ^a^ Calculated as weight in kilograms divided by height in meters squared

Supplemental Table 4 Correlations of night sleep duration with cognitive performance in Chinese elderly persons at each time point from 2011 to 2018.

|  | NS2011 | NS2013 | NS2015 | NS2018 | Cog2011 | Cog2013 | Cog2015 | Cog2018 |
| --- | --- | --- | --- | --- | --- | --- | --- | --- |
| NS2011 | 1 |  |  |  |  |  |  |  |
| NS2013 | 0.407^a^ | 1 |  |  |  |  |  |  |
| NS2015 | 0.398 ^a^ | 0.446^a^ | 1 |  |  |  |  |  |
| NS2018 | 0.348^a^ | 0.379^a^ | 0.422^a^ | 1 |  |  |  |  |
| Cog2011 | 0.099^a^ | 0.068^a^ | 0.062^a^ | 0.059^a^ | 1 |  |  |  |
| Cog2013 | 0.089^a^ | 0.060^a^ | 0.051^a^ | 0.028^a^ | 0.593^a^ | 1 |  |  |
| Cog2015 | 0.090^a^ | 0.059^a^ | 0.054^a^ | 0.029^a^ | 0.636^a^ | 0.662^a^ | 1 |  |
| Cog2018 | 0.086^a^ | 0.042^a^ | 0.026^b^ | 0.014 | 0.611^a^ | 0.635^a^ | 0.706^a^ | 1 |

Abbreviations: NS Night sleep duration, Cog Cognition.

^a^ *P*< 0.01

^b^ *P*< 0.05

Supplemental Table 5 Correlations of night sleep duration with each cognitive domain performance in Chinese elderly persons at each time point during 2011-2018.

|  | NS2011 | NS2013 | NS2015 | NS2018 | NP2011 | NP2013 | NP2015 | NP2018 | Ori2011 | Ori2013 | Ori2015 | Ori2018 | Mem2011 | Mem2013 | Mem2015 | Mem2018 | Exe2011 | Exe2013 | Exe2015 | Exe2018 |
| --- | --- | --- | --- | --- | --- | --- | --- | --- | --- | --- | --- | --- | --- | --- | --- | --- | --- | --- | --- | --- |
| NS2011 | 1 |  |  |  |  |  |  |  |  |  |  |  |  |  |  |  |  |  |  |  |
| NS2013 | 0.407^a^ | 1 |  |  |  |  |  |  |  |  |  |  |  |  |  |  |  |  |  |  |
| NS2015 | 0.398 ^a^ | 0.446^a^ | 1 |  |  |  |  |  |  |  |  |  |  |  |  |  |  |  |  |  |
| NS2018 | 0.348^a^ | 0.379^a^ | 0.422^a^ | 1 |  |  |  |  |  |  |  |  |  |  |  |  |  |  |  |  |
| NP2011 | 0.109^a^ | 0.08^a^ | 0.088^a^ | 0.083^a^ | 1 |  |  |  |  |  |  |  |  |  |  |  |  |  |  |  |
| NP2013 | 0.09^a^ | 0.078^a^ | 0.09^a^ | 0.069^a^ | 0.360^a^ | 1 |  |  |  |  |  |  |  |  |  |  |  |  |  |  |
| NP2015 | 0.077^a^ | 0.087^a^ | 0.106^a^ | 0.084^a^ | 0.436^a^ | 0.469^a^ | 1 |  |  |  |  |  |  |  |  |  |  |  |  |  |
| NP2018 | 0.058^a^ | 0.078^a^ | 0.082^a^ | 0.107^a^ | 0.267^a^ | 0.297^a^ | 0.343^a^ | 1 |  |  |  |  |  |  |  |  |  |  |  |  |
| Ori2011 | 0.063^a^ | 0.038^a^ | 0.049^a^ | 0.041^a^ | 0.023^b^ | 0.033^a^ | 0.022^b^ | -0.019 | 1 |  |  |  |  |  |  |  |  |  |  |  |
| Ori2013 | 0.056^a^ | 0.05^a^ | 0.034^a^ | 0.019 | 0.024^b^ | 0.012 | 0 | -0.019 | 0.496^a^ | 1 |  |  |  |  |  |  |  |  |  |  |
| Ori2015 | 0.069^a^ | 0.036^a^ | 0.045^a^ | 0.033^a^ | 0.039^a^ | 0.038^a^ | 0.004 | -0.004 | 0.463^a^ | 0.496^a^ | 1 |  |  |  |  |  |  |  |  |  |
| Ori2018 | 0.057^a^ | 0.021^b^ | 0.01 | -0.002 | 0.026^b^ | 0.017 | -0.1 | -0.035^a^ | 0.433^a^ | 0.468^a^ | 0.525^a^ | 1 |  |  |  |  |  |  |  |  |
| Mem2011 | 0.076^a^ | 0.051^a^ | 0.040^a^ | 0.033^a^ | 0.018 | -0.001 | -0.009 | -0.006 | 0.339^a^ | 0.268^a^ | 0.293^a^ | 0.300^a^ | 1 |  |  |  |  |  |  |  |
| Mem2013 | 0.063^a^ | 0.032^a^ | 0.032^a^ | 0.001 | -0.001 | -0.009 | -0.1 | -0.027^a^ | 0.312^a^ | 0.438^a^ | 0.345^a^ | 0.359^a^ | 0.398^a^ | 1 |  |  |  |  |  |  |
| Mem2015 | 0.070^a^ | 0.038^a^ | 0.039^a^ | 0.003 | 0.019 | -0.005 | -0.013 | -0.036^a^ | 0.331^a^ | 0.361^a^ | 0.441^a^ | 0.407^a^ | 0.440^a^ | 0.520^a^ | 1 |  |  |  |  |  |
| Mem2018 | 0.083^a^ | 0.039^b^ | 0.022^b^ | 0.004 | 0.044^a^ | 0.025^a^ | -0.004 | -0.019 | 0.372^a^ | 0.378^a^ | 0.423^a^ | 0.526^a^ | 0.407^a^ | 0.484^a^ | 0.555^a^ | 1 |  |  |  |  |
| Exe2011 | 0.089^a^ | 0.068^a^ | 0.054^a^ | 0.063^a^ | 0.054^a^ | 0.059^a^ | 0.040^a^ | 0.021^b^ | 0.459^a^ | 0.378^a^ | 0.416^a^ | 0.401^a^ | 0.394^a^ | 0.343^a^ | 0.369^a^ | 0.423^a^ | 1 |  |  |  |
| Exe2013 | 0.098^a^ | 0.062^a^ | 0.058^a^ | 0.048^a^ | 0.070^a^ | 0.049^a^ | 0.038^a^ | 0.013 | 0.369^a^ | 0.502^a^ | 0.414^a^ | 0.423^a^ | 0.305^a^ | 0.483^a^ | 0.401^a^ | 0.452^a^ | 0.521^a^ | 1 |  |  |
| Exe2015 | 0.078^a^ | 0.068^a^ | 0.046^a^ | 0.032^a^ | 0.051^a^ | 0.055^a^ | 0.025^b^ | 0.014 | 0.383^a^ | 0.396^a^ | 0.501^a^ | 0.449^a^ | 0.323^a^ | 0.374^a^ | 0.456^a^ | 0.474^a^ | 0.540^a^ | 0.556^a^ | 1 |  |
| Exe2018 | 0.073^a^ | 0.045^a^ | 0.032^b^ | 0.034^a^ | 0.062^a^ | 0.048^a^ | 0.014 | -0.006 | 0.371^a^ | 0.377^a^ | 0.424^a^ | 0.513^a^ | 0.325^a^ | 0.378^a^ | 0.432^a^ | 0.577^a^ | 0.508^a^ | 0.526^a^ | 0.561^a^ | 1 |

Abbreviations: NS Night sleep duration, Ori Orientation, Mem Memory, Exe Executive function.

^a^ *P*< 0.01

^b^ *P*< 0.05

Supplemental Table 6 Levels of cognitive performance and sleep duration in elderly Chinese participants at each time point.

| Variable | Wave 1(2011) | | | Wave 2(2013) | | | Wave 3(2015) | | | Wave 4(2018) | | |
| --- | --- | --- | --- | --- | --- | --- | --- | --- | --- | --- | --- | --- |
|  | No. of participants | Mean±SD | p value | No. of participants | Mean±SD | p value | No. of participants | Mean±SD | p value | No. of participants | Mean±SD | p value |
| Cognition score | F=564.984, p<0.001 | | | | | | | | | | | |
| Total | 9404 | 13.84±6.31 |  | 9404 | 13.66±6.71 |  | 9404 | 13.07±6.59 |  | 9404 | 11.37±7.69 |  |
| Age, years |  |  | <0.001 |  |  | <0.001 |  |  | <0.001 |  |  | <0.001 |
| 45-59 | 5376 | 14.95±6.06 |  | 5376 | 14.93±6.41 |  | 5376 | 14.65±6.19 |  | 5376 | 13.21±7.44 |  |
| 60-64 | 1763 | 13.36±6.16 |  | 1763 | 13.40±6.36 |  | 1763 | 12.71±6.23 |  | 1763 | 10.73±7.26 |  |
| 65-79 | 2111 | 11.71±6.25 |  | 2111 | 11.20±6.72 |  | 2111 | 10.17±6.49 |  | 2111 | 7.89±7.08 |  |
| 80+ | 154 | 7.63±6.11 |  | 154 | 6.29±6.32 |  | 154 | 4.84±5.66 |  | 154 | 2.19±4.44 |  |
| Gender |  |  | <0.001 |  |  | <0.001 |  |  | <0.001 |  |  | <0.001 |
| Male | 4338 | 15.21±5.83 |  | 4338 | 14.97±6.33 |  | 4338 | 14.36±6.08 |  | 4338 | 12.72±7..35 |  |
| Female | 5066 | 12.68±6.47 |  | 5066 | 12.55±6.83 |  | 5066 | 11.97±6.81 |  | 5066 | 10.21±7.79 |  |
| Night Sleep duration | F=47.02, p<0.001 | | | | | | | | | | | |
| Total | 9404 | 6.37±1.86 |  | 9404 | 6.16±1.84 |  | 9404 | 6.32±2.00 |  | 9404 | 6.18±2.13 |  |
| Age, years |  |  | <0.001 |  |  | <0.001 |  |  | <0.001 |  |  | 0.595 |
| 45-59 | 5376 | 6.52±1.75 |  | 5376 | 6.22±1.73 |  | 5376 | 6.38±1.86 |  | 5376 | 6.20±1.92 |  |
| 60-64 | 1763 | 6.24±1.84 |  | 1763 | 6.09±1.87 |  | 1763 | 6.33±2.03 |  | 1763 | 6.15±2.19 |  |
| 65-79 | 2111 | 6.10±2.07 |  | 2111 | 6.04±2.05 |  | 2111 | 6.17±2.23 |  | 2111 | 6.14±2.48 |  |
| 80+ | 154 | 6.16±2.27 |  | 154 | 6.27±2.31 |  | 154 | 6.24±2.59 |  | 154 | 6.45±2.92 |  |
| Gender |  |  | <0.001 |  |  | <0.001 |  |  | <0.001 |  |  | <0.001 |
| Male | 4338 | 6.50±1.76 |  | 4338 | 6.33±1.72 |  | 4338 | 6.52±1.82 |  | 4338 | 6.44±2.03 |  |
| Female | 5066 | 6.25±1.93 |  | 5066 | 6.01±1.93 |  | 5066 | 6.15±2.12 |  | 5066 | 5.95±2.18 |  |

^a^ *P* value was calculated by one-way analysis of variance (ANOVA).

^b^ *P* value was calculated by one-way repeated measures ANOVA.

Supplemental Table 7. Adjust β Coefficients for the Random Intercept Cross-Lagged Panel Model Examining the Relationships Between Night Sleep duration and Cognitions.

|  | β (95% CI) | | | | | | | | |  | |
| --- | --- | --- | --- | --- | --- | --- | --- | --- | --- | --- | --- |
| Predict variables | RI_Ns (short)^a^ | | RI_Ns (moderate)^b^ | | | RI_Ns (long)^c^ | | RI_Ns^d^ | | RI_Cog^e^ | |
| Age | -0.015 (-0.017, -0.013)^*g^ | | -0.006 (-0.008, -0.004)^*^ | | | 0.002 (-0.001, 0.005) | | -0.01 (-0.012, -0.008)^*g^ | | -0.034 (-0.035, -0.035)^*g^ | |
| Gender | -0.142 (-0.185, -0.099)^*g^ | | -0.062 (-0.093, -0.031)^*^ | | | -0.053 (-0.113, 0.007)^g^ | | -0.117 (-0.148, -0.086)^*g^ | | -0.468 (-0.487, -0.449)^*g^ | |
| High level of education | 0.237 (0.269, 0.302)^*g^ | | -0.025 (-0.068, 0.018) | | | -0.124 (-0.228, -0.02)^g^ | | -0.019 (-0.063, 0.025) | | 0.667 (0.64, 0.694)^*g^ | |
| Taking sleeping pills | -0.124 (-0.504, 0.256) | | -0.238 (-0.915, 0.441) | | | -0.956 (-1.673, -0.239)^g^ | | -0.497 (-0.877, -0.117)^g^ | | -0.113 (-0.346, 0.12) | |
| Hypertension | 0.052 (0.006, 0.098)^g^ | | -0.038 (-0.073, -0.003)g | | | 0.08 (0.014, 0.146)^g^ | | -0.025 (-0.059, 0.009)^g^ | | 0.014 (-0.007, 0.035)^g^ | |
| Dyslipidemia | 0.005 (-0.062, 0.072) | | 0.012 (-0.038, 0.062) | | | -0.042 (-0.145, 0.061) | | 0.025 (-0.023, 0.073) | | -0.126 (-0.156, -0.096)^*g^ | |
| Diabetes | -0.116 (-0.198, -0.034)g | | 0.044 (-0.017, 0.107) | | | -0.082 (-0.208, 0.044) | | -0.027 (-0.086, -0.032) | | -0.038 (-0.074, -0.002) | |
| Cancer | 0.018 (-0.158, 0.194) | | 0.26 (0.109, 0.411)^g^ | | | 0.379 (0.11, 0.648)^g^ | | 0.244 (0.107, 0.381)^g^ | | 0.099 (0.015, 0.183)^g^ | |
| Chronic lung diseases | 0.136 (0.075, 0.197)^*g^ | | -0.027 (-0.077, 0.023) | | | 0.111 (0.001, 0.221)^g^ | | 0.113 (0.066, 0.16)^*g^ | | 0.018 (-0.011, 0.047)^g^ | |
| Liver disease | -0.097 (-0.18, -0.016)^g^ | | 0.013 (-0.06, 0.086) | | | -0.257 (-0.428, -0.086)^g^ | | 0.07 (0.003, 0.137)^g^ | | -0.052 (-0.093, 0.011) | |
| Heart problems | -0.05 (-0.109, -0.009)^g^ | | 0.007 (-0.038, 0.052) | | | 0.018 (-0.079, 0.115) | | 0.002 (-0.042, 0.046) | | -0.129 (-0.156, -0.102)^*g^ | |
| Stroke | 0.018 (-0.102, 0.138) | | -0.116 (-0.219, -0.013)^g^ | | | 0.047 (-0.169, 0.263) | | 0.02 (-0.074, 0.114) | | 0.102 (0.044, 0.16)^g^ | |
| Kidney disease | 0.048 (-0.024, 0.12) | | 0.122 (0.063, 0.181)^*g^ | | | 0.086 (-0.024, 0.196)^g^ | | 0.045 (-0.01, 0.1)^g^ | | 0.03 (-0.004, 0.064) | |
| Stomach or other digestive disease | 0.138 (0.095, 0.181)^*g^ | | 0.091 (0.058, 0.124)^*g^ | | | -0.002 (-0.067, 0.063) | | 0.096 (0.064, 0.128)^*g^ | | 0.023 (0.004, 0.042)^g^ | |
| Emotional, nervous, or psychiatric problems | 0.217 (-0.187, 0.621) | | -0.013 (-0.789, 0.763) | | | -1.151 (-1.906, -0.306) | | -0.301 (-0.692, -0.09)^g^ | | 0.162 (-0.078, 0.302)^g^ | |
| Memory-related disease | 0.217 (0.037, 0.387)^g^ | | -0.14 (-0.276, -0.004)^g^ | | | -0.764 (-1.006, -0.522)^*g^ | | -0.169 (-0.296, -0.042)^g^ | | 0.244 (0.166, 0.322)^*g^ | |
| Arthritis or rheumatism | 0.095 (0.057, 0.133)^*g^ | | 0.081 (0.051, 0.111)^*g^ | | | -0.02 (-0.079, 0.039) | | 0.182 (0.154, 0.21)^*g^ | | 0.078 (0.061, 0.095)^*g^ | |
| Asthma | 0.122 (0.028, 0.216)^g^ | | 0.086 (-0.003, 0.169)^g^ | | | -0.166 (-0.321, -0.011)^g^ | | 0.027 (-0.048, 0.102) | | 0.032 (-0.014, 0.078) | |
| Napping at baseline | 0.084 (0.057, 0.111)^*g^ | | 0.112 (0.093, 0.131)^*g^ | | | 0.097 (0.064, 0.13)^*g^ | | 0.151 (0.133, 0.169)^*g^ | | 0.007 (-0.004, 0.018) | |
| Self-reported sleep quality at baseline | -0.476 (-0.491, -0.461)^*g^ | | -0.345 (-0.358, -0.332)^*g^ | | | -0.042 (-0.07, -0.014)^g^ | | -0.536 (-0.547, -0.525)^*g^ | | -0.056 (-0.063, -0.049)^*g^ | |
| Depression at baseline | -0.104 (-0.14, -0.068)^*g^ | | 0.016 (-0.011, 0.043) | | | -0.015 (-0.066, 0.036) | | -0.031 (-0.057, -0.005)^g^ | | -0.055 (-0.071, -0.039)^*g^ | |
| BMI at baseline | 0.015 (0.01, 0.02)^*g^ | | 0.003 (-0.001, 0.007)^g^ | | | 0.002 (-0.005, 0.009) | | 0.007 (0.003, 0.011)^*g^ | | 0.021 (0.019, 0.023)^*g^ | |
| Smoking at baseline | 0.104 (0.065, 0.143)^*g^ | | 0.062 (0.034, 0.09)^*g^ | | | 0.142 (0.087, 0.197)^*g^ | | 0.131 (0.103, 0.159)^*g^ | | -0.167 (-0.184, -0.15)^*g^ | |
| Current drinking at baseline | -0.081 (-0.128, -0.034)^g^ | | -0.076 (-0.11, -0.042)^*g^ | | | 0.029 (-0.037, 0.095)^g^ | | -0.087 (-0.12, -0.054)^*g^ | | -0.011 (-0.031, 0.009) | |
| Vigorous physical activities at baseline | 0.051 (0.007, 0.095)^g^ | | -0.019 (-0.051, 0.013) | | | -0.031 (-0.095, 0.031)^g^ | | -0.009 (-0.04, -0.022) | | -0.131 (-0.15, -0.112)^*g^ | |
| Moderate physical activities at baseline | 0.023 (-0.022, 0.068) | | -0.049 (-0.082, -0.016)^g^ | | | -0.148 (-0.217, -0.079)^*g^ | | -0.027 (-0.059, 0.005)^g^ | | 0.032 (0.012, 0.052)^g^ | |
| Walking at baseline | 0.057 (0.017, 0.097)^g^ | | 0.028 (-0.002, 0.058)^g^ | | | 0.09 (0.028, 0.152)^g^ | | 0.034 (0.005, 0.063)^g^ | | 0.075 (0.057, 0.093)^*g^ | |
|  | | β (95% CI)^f^ | | | | | | | | |  |
| Path | | Short^h^ (<=6h/d) | | | Moderate^i^ (6-8h/d) | | Long^j^ (>8h/d) | | Night sleep duration | |  |
| RIns^k^~Rico^l^ | | 0.87 (0.071, 0.103)^*g^ | | | -0.014 (-0.026, -0.002)^g^ | | -0.06 (-0.083, -0.037)^*g^ | | -0.008 (-0.019, 0.003)^g^ | |  |
| Stability paths | |  | | |  | |  | |  | |  |
| W1 sleep duration → W2 sleep duration | | -0.179 (-0.232, -0.126)^*g^ | | | -0.109 (-0.16, -0.058)^*g^ | | -0.036 (-0.229, 0.157) | | -0.059 (-0.077, -0.041)^*g^ | |  |
| W2 sleep duration →W3 sleep duration | | 0.223 (0.201, 0.245)^*g^ | | | 0.277 (0.259, 0.295)^*g^ | | 0.295 (0.243, 0.337)^*g^ | | 0.159 (0.143, 0.175)^*g^ | |  |
| W3 sleep duration →W4 sleep duration | | 0.231 (0.211, 0.251)^*g^ | | | 0.28 (0.264, 0.296)^*g^ | | 0.311 (0.272, 0.35)^*g^ | | 0.186 (0.173, 0.199)^*g^ | |  |
| W1 cognition → W2 cognition | | -0.114 (-0.152, -0.076)^*g^ | | | -0.103 (-0.134, -0.072)^*g^ | | -0.195 (-0.275, -0.115)^*g^ | | -0.125 (-0.148, -0.102)^*g^ | |  |
| W2 cognition → W3 cognition | | 0.106 (0.08, 0.132)^*g^ | | | 0.107 (0.087, 0.127)^*g^ | | 0.139 (0.087, 0.191)^*g^ | | 0.102 (0.087, 0.117)^*g^ | |  |
| W3 cognition → W4 cognition | | 0.288 (0.263, 0.313)^*g^ | | | 0.305 (0.284, 0.326)^*g^ | | 0.169 (0.117, 0.221)^*g^ | | 0.283 (0.267, 0.299)^*g^ | |  |
| Cross-Lagged paths | |  | | |  | |  | |  | |  |
| W1 sleep duration → W2 cognition | | 0.059 (0.038, 0.08)^*g^ | | | -0.009 (-0.029, 0.011) | | 0.072 (0.011, 0.133)^g^ | | 0.008 (0, 0.016) | |  |
| W2 sleep duration → W3 cognition | | -0.021 (-0.031, -0.011)^*g^ | | | 0.013 (0.004, 0.022) | | 0.024 (0.007, 0.041)^g^ | | 0.011 (0.004, 0.018) | |  |
| W3 sleep duration → W4 cognition | | -0.04 (-0.048, -0.032)^*g^ | | | -0.017 (-0.024, -0.01)^*g^ | | 0.013 (-0.001, 0.027) | | -0.021 (-0.027, 0.015)^*g^ | |  |
| W1 Cognition →W2 seep duration | | -0.165 (-0.241, -0.089)^*g^ | | | 0.057 (0, 0.114) | | 0.53 (0.363, 0.697)^*g^ | | 0.032 (-0.011, 0.075) | |  |
| W2 cognition →W3 sleep duration | | -0.099 (-0.154, -0.044)^g^ | | | 0.036 (-0.003, 0.075) | | 0.022 (-0.105, 0.149) | | 0.025 (-0.007, 0.057) | |  |
| W3 cognition →W4 sleep duration | | -0.259 (-0.316, -0.202)^*g^ | | -0.011 (-0.057, 0.035) | | | 0.25 (0.112, 0.288)^g^ | | -0.061 (-0.096, -0.026)^g^ | |  |

Note: ^a^ RI_Ns(short), random intercept of short night sleep duration (≤6 hours/day).

^b^ RI_Ns(moderate), random intercept of short night sleep duration (6-8 hours/day).

^c^ RI_Ns(long), random intercept of short night sleep duration (>8 hours/day).

^d^ RI_Ns, random intercept of night sleep duration.

^e^ RI_Cog, random intercept of cognitive performance z score

^f^ Adjust coefficients are presented in these models. The subscript numbers indicate the wave of the study (wave1, wave2, wave3 and wave 4).

* Significant at *P*<0.05

^g^ Significant at *P FDR*<0.05

^h^ Short, participants with short night duration, slept <=6 hours per day.

^i^ Moderate, participants with moderate night duration, slept 6-8 hours per day.

^j^ Long, participants with long night duration, slept >8 hours per day.

^k^ RIns, random intercept of night sleep^g^ RIco, random intercept of overall cognitive performance

^l^ RIco, random intercept of overall cognitive performance

Supplemental Table 8. Standardized β Coefficients for the Random Intercept Cross-Lagged Panel Model Examining the Relationships Between Night Sleep Duration and Cognitions in male and female^a^.

|  | β (95% CI) | |
| --- | --- | --- |
| Path | Male | Female |
| RIns~RIco | **0.044 (0.026, 0.062)^*b^** | **0.132 (0.11, 0.154)^*b^** |
| Stability paths |  |  |
| W1 sleep duration → W2 sleep duration | -0.045 (-0.069, -0.021) | -0.013 (-0.036, 0.01) |
| W2 sleep duration →W3 sleep duration | **0.08 (0.055, 0.105)^*b^** | **0.090 (0.066, 0.114)^*b^** |
| W3 sleep duration →W4 sleep duration | **0.117 (0.094, 0.14)^*b^** | 0.142 (0.123, 0.161) |
| W1 cognition → W2 cognition | **-0.227 (-0.267, -0.187)^*b^** | **-0.328 (-0.374, 0.282)^*b^** |
| W2 cognition → W3 cognition | **0.098 (0.076, 0.121)^*b^** | **0.101 (0.076, 0.126)^*b^** |
| W3 cognition → W4 cognition | **0.338 (0.316, 0.361)^*b^** | **0.354 (0.334, 0.374)^*b^** |
| Cross-Lagged paths |  |  |
| W1 sleep duration → W2 cognition | 0.014 (0.001, 0.027) | **0.025 (0.014, 0.036)^*b^** |
| W2 sleep duration → W3 cognition | -0.002 (-0.014, 0.01) | -0.003 (-0.014,0.008) |
| W3 sleep duration → W4 cognition | **-0.038 (-0.048, -0.028)^*b^** | **-0.018 (-0.025, -0.011)^*b^** |
| W1 cognition →W2 sleep duration | 0.034 (-0.03, 0.098) | 0.063 (-0.014, 0.140) |
| W2 cognition →W3 sleep duration | 0.032 (-0.011, 0.075) | -0.061 (-0.113, -0.009) |
| W3 cognition →W4 sleep duration | **-0.154 (-0.203, -0.105)^*b^** | -0.087 (-0.134, -0.04) |

Note: W1, wave 1 (CHARLS 2011 survey); W2, wave 2 (CHARLS 2013 survey); W3, wave 3 (CHARLS 2015 survey); W4, wave 4 (CHARLS 2018 survey).

^a^ Standardized coefficients are presented in these models. The subscript numbers indicate the wave of the study (wave1, wave2, wave3 and wave 4).

*Significant at *P*<0.05

^b^ Significant at *P FDR*<0.05

^c^ RIns, random intercept of night sleep

^d^ RIco, random intercept of overall cognitive performance

Supplemental Table 9. Standardized β Coefficients for the Random Intercept Cross-Lagged Panel Model Examining the Relationships Between Night Sleep Duration and Cognitions in different educational levels^a^.

|  | β (95% CI) | |
| --- | --- | --- |
| Path | Low educational level | High educational level |
| RIns^c^~RIco^d^ | **0.117 (0.102, 0.132)^*b^** | 0.036 (0.014, 0.058) |
| Stability paths |  |  |
| W1 sleep duration → W2 sleep duration | **-0.036 (-0.054, -0.018)^*^** | -0.067 (0.012, 0.122) |
| W2 sleep duration →W3 sleep duration | **0.088 (0.070, 0.106)^*b^** | 0.078 (0.019, 0.137) |
| W3 sleep duration →W4 sleep duration | **0.117 (0.094, 0.14)^*b^** | 0.174 (0.130, 0.218) |
| W1 cognition → W2 cognition | **-0.248 (-0.278, -0.218)^*b^** | -0.053 (-0.119, 0.13) |
| W2 cognition → W3 cognition | **0.104 (0.086, 0.122)^*b^** | 0.048 (0.014, 0.082) |
| W3 cognition → W4 cognition | **0.322 (0.306, 0.338)^*b^** | **0.295 (0.247, 0.343)^*b^** |
| Cross-Lagged paths |  |  |
| W1 sleep duration → W2 cognition | 0.016 (0.007, 0.025) | -0.006 (-0.041, 0.029) |
| W2 sleep duration → W3 cognition | 0.001 (-0.007, 0.009) | **-0.058 (-0.086,-0.030)^*^** |
| W3 sleep duration → W4 cognition | **-0.022 (-0.028, -0.016)^*b^** | **-0.060 (-0.084, -0.036)^*b^** |
| W1 cognition →W2 sleep duration | 0.043 (-0.09, 0.095) | -0.028(-0.118, 0.062) |
| W2 cognition →W3 sleep duration | -0.011 (-0.049, 0.027) | -0.019 (-0.084, -0.046) |
| W3 cognition →W4 sleep duration | **-0.112 (-0.149, -0.075)^*b^** | -0.033 (-0.118, -0.052) |

Note: W1, wave 1 (CHARLS 2011 survey); W2, wave 2 (CHARLS 2013 survey); W3, wave 3 (CHARLS 2015 survey); W4, wave 4 (CHARLS 2018 survey).

^a^ Standardized coefficients are presented in these models. The subscript numbers indicate the wave of the study (wave1, wave2, wave3 and wave 4).

*Significant at *P*<0.05

^b^ Significant at *P FDR*<0.05

^c^ RIns, random intercept of night sleep^g^ RIco, random intercept of overall cognitive performance

^d^ RIco, random intercept of overall cognitive performance

Supplemental Table 10. Standardized β Coefficients for the Random Intercept Cross-Lagged Panel Model Examining the Relationships Between Night Sleep Duration and Cognitions in specific cognitive domains^a^.

|  | β (95% CI) | | | |
| --- | --- | --- | --- | --- |
| Path | Orientation | Memory | Executive function | Overall Cognition |
| RIns^c^~RIco^d^ | **0.089 (0.061, 0.117) ^*b^** | **0.090 (0.074, 0.116) ^*b^** | **0.116 (0.080, 0.142)^*b^** | **0.13 (0.115, 0.145)^*b^** |
| Stability paths |  |  |  |  |
| W1 sleep duration → W2 sleep duration | -0.028 (-0.045, -0.011) | **-0.034 (-0.051, -0.017)^*^** | -0.030 (-0.047, -0.013) | -0.031 (-0.048, -0.014) |
| W2 sleep duration →W3 sleep duration | **0.077 (0.059, 0.95)^*b^** | **0.074 (0.066, 0.092)^*b^** | **0.076 (0.058, 0.094)^*b^** | **0.087 (0.069, 0.105)^*b^** |
| W3 sleep duration →W4 sleep duration | **0.138 (0.124, 0.152)^*b^** | **0.125 (0.112, 0.139)^*b^** | **0.123 (0.109, 0.137)^*b^** | **0.139 (0.125, 0.153)^*b^** |
| W1 cognition → W2 cognition | -0.007 (-0.026, 0.012) | **-0.189 (-0.207, -0.171)^*b^** | -0.012 (-0.029, 0.005) | **-0.279 (-0.310, -0.248)^*b^** |
| W2 cognition → W3 cognition | **0.124 (0.110, 0.138)^*b^** | **0.054 (0.036, 0.072)^*b^** | **0.045 (0.029, 0.061)^*b^** | **0.097 (0.08, 0.116)^*b^** |
| W3 cognition → W4 cognition | **0.149 (0.135, 0.163)^*b^** | **0.236 (0.219, 0.253)^*b^** | **0.070 (0.054, 0.086)^*b^** | **0.344 (0.329, 0.359)^*b^** |
| Cross-Lagged paths |  |  |  |  |
| W1 sleep duration → W2 cognition | 0.013 (0.004, 0.022) | 0.019 (0.011, 0.027) | **0.028 (0.012, 0.044) ^*b^** | 0.016 (0.008, 0.024) |
| W2 sleep duration → W3 cognition | -0.018 (-0.027, -0.009) | -0.019 (-0.027, -0.011) | 0.008 (-0.008, 0.024) | -0.002 (-0.01, 0.006) |
| W3 sleep duration → W4 cognition | **-0.025 (-0.032, -0.018)^*b^** | **-0.036 (-0.043, -0.029)^*b^** | **-0.016 (-0.028, -0.004)^*b^** | **-0.025 (-0.031, -0.019)^*b^** |
| W1 cognition →W2 sleep duration | -0.004 (-0.035, 0.027) | 0.011 (-0.021, 0043) | 0.045 (-0.019, 0.077) | 0.037 (-0.014, 0.088) |
| W2 cognition →W3 sleep duration | -0.011 (-0.037, 0.015) | -0.008 (-0.042, 0.026) | 0.008 (-0.026, 0.042) | -0.017 (-0.051, 0.017) |
| W3 cognition →W4 sleep duration | -0.011 (-0.039, 0.017) | **-0.042 (-0.073, -0.011)^*b^** | **-0.114 (-0.184, -0.044)^*b^** | **-0.103 (-0.137, -0.069)^*b^** |

Note: W1, wave 1 (CHARLS 2011 survey); W2, wave 2 (CHARLS 2013 survey); W3, wave 3 (CHARLS 2015 survey); W4, wave 4 (CHARLS 2018 survey).

^a^ Standardized coefficients are presented in these models. The subscript numbers indicate the wave of the study (wave1, wave2, wave3 and wave 4).

*Significant at *P*<0.05

^b^ Significant at *P FDR*<0.05

^c^ RIns, random intercept of night sleep

^d^ RIco, random intercept of cognitive performance (overall, orientation, executive function, and memory)

Supplemental Table 11 Standardized β Coefficients for the Random Intercept Cross-Lagged Panel Model Examining the Relationships Between Night Sleep duration and Cognitions by using complete data from participants without missing variables^a^

|  | β (95% CI) | | | |
| --- | --- | --- | --- | --- |
| Path | Short^c^ (<=6h/d) | Moderate^d^ (6-8h/d) | Long^e^ (>8h/d) | Night sleep duration |
| RIns^f^~RIco^g^ | **1.085 (0.773, 1.397)^*b^** | -0.045 (-0.193, 0.103) | **-0.722 (-1.226, -0. 218)^*^** | **0.623 (0.365, 0.881)^*b^** |
| Stability paths |  |  |  |  |
| W1 sleep duration → W2 sleep duration | **-0.261 (-0.451, -0.071)^*b^** | -0.359 (-0.915, -0.197) | -0.042 (-1.170, 0.330) | -0.044 (-0.010, 0.012) |
| W2 sleep duration →W3 sleep duration | **0.240 (0.174, 0.306)^*b^** | **0.353 (0.295, 0.411)^*b^** | **0.268 (0.118, 0.418)^*b^** | **0.082 (0.022, 0.142)^*b^** |
| W3 sleep duration →W4 sleep duration | **0.233 (0.173, 0.293)^*b^** | **0.381 (0.323, 0.440)^*b^** | **0.237 (0.101, 0.373)^*b^** | **0.125 (0.073, 0.177)^*b^** |
| W1 cognition → W2 cognition | **-0.189 (-0.301, -0.077)^*b^** | **-0.201 (-0.315, -0.087)^*b^** | -0.199 (-0.473, 0.075) | **-0.217 (-0.295, -0.139)^*b^** |
| W2 cognition → W3 cognition | 0.089 (-0.011, 0.189) | 0.076 (-0.038, 0.190) | 0.103 (-0.157, 0.363) | **0.085 (0.013, 0.157)^*b^** |
| W3 cognition → W4 cognition | **0.399 (0.313, 0.485)^*b^** | **0.412 (0.322, 0.502)^*b^** | 0.229 (0.019, 0.439)^*^ | **0.392 (0.332, 0.452)^*b^** |
| Cross-Lagged paths |  |  |  |  |
| W1 sleep duration → W2 cognition | 0.286 (-0.176, 0.748) | -0.052 (-0.156, 0.052) | 0.778 (-0.556, 2.112) | **0.218 (0.054, 0.382)^*b^** |
| W2 sleep duration → W3 cognition | -0.162 (-0.362, -0.038) | 0.004 (-0.204, 0.212) | 0.517 (0.085, 0.949)^*^ | -0.016 (-0.188, 0.156) |
| W3 sleep duration → W4 cognition | **-0.363 (-0.543, -0.183)^*b^** | -0.110 (-0.302, -0.082) | 0.153 (-0.229, 0.535) | **-0.243 (-0.395, -0.091)^*b^** |
| W1 Cognition →W2 seep duration | -0.020 (-0.056, -0.016) | 0.004 (-0.030, 0.038) | 0.100 (0.006, 0.194)^*^ | 0.002 (-0.022, 0.026) |
| W2 cognition →W3 sleep duration | -0.017 (-0.049, 0.015) | 0.005 (-0.027, 0.037) | 0.045 (-0.047, 0.137) | 0.006 (-0.016, 0.028) |
| W3 cognition →W4 sleep duration | -0.026 (-0.054, 0.002) | -0.001 (-0.029, 0.027) | 0.035 (-0.037, 0.107) | -0.017 (-0.037, 0.003) |

Note: W1, wave 1 (CHARLS 2011 survey); W2, wave 2 (CHARLS 2013 survey); W3, wave 3 (CHARLS 2015 survey); W4, wave 4 (CHARLS 2018 survey).

^a^ Standardized coefficients are presented in these models. The subscript numbers indicate the waves of the study (wave1, wave 2, wave 3 and wave 4).

*Significant at *P*<0.05

^b^ Significant at *P FDR*<0.05

^c^ Short, participants with short night duration, slept <=6 hours per day.

^d^ Moderate, participants with moderate night duration, slept 6-8 hours per day.

^e^ Long, participants with long night duration, slept >8 hours per day.

^f^ RIns, random intercept of night sleep^g^ RIco, random intercept of overall cognitive performance

^g^ RIco, random intercept of overall cognitive performance


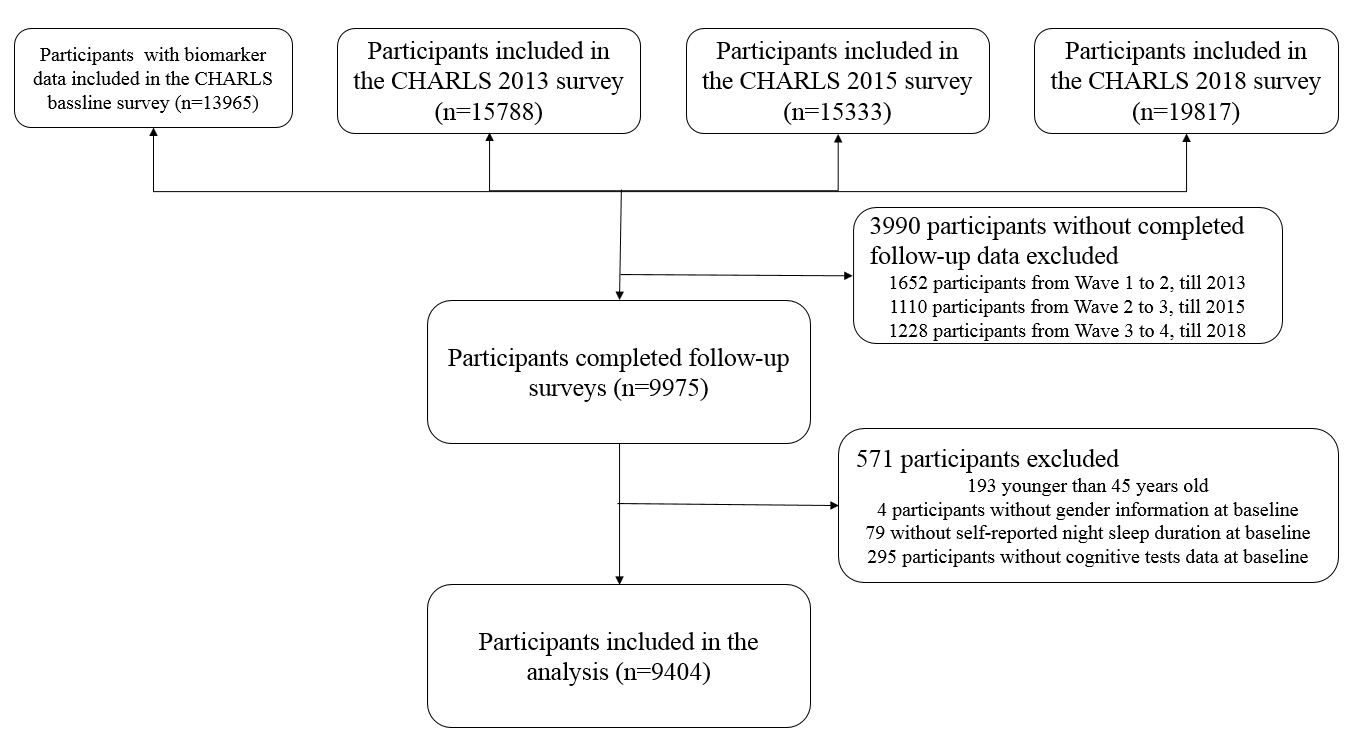


Supplemental Figure 1. Flow chart of participant selection for this study.
